# Associations of sex hormone-binding globulin and testosterone with genome-wide DNA methylation

**DOI:** 10.1186/s12863-018-0703-y

**Published:** 2018-12-14

**Authors:** Ryan Arathimos, Gemma C. Sharp, Raquel Granell, Kate Tilling, Caroline L. Relton

**Affiliations:** 1 Population Health Sciences, Bristol Medical School, University of Bristol, Bristol, UK; 2 Medical Research Council Integrative Epidemiology Unit, University of Bristol, Oakfield House, Bristol, BS8 2BN UK; 3 Bristol Dental School, University of Bristol, Bristol, UK

**Keywords:** Sex hormones, DNA methylation, Epigenetics, Testosterone, SHBG, ALSPAC

## Abstract

**Background:**

Levels of sex hormone-binding globulin (SHBG) and the androgen testosterone have been associated with risk of diseases throughout the lifecourse. Although both SHBG and testosterone have been shown to be highly heritable, only a fraction of that heritability has been explained by genetic studies. Epigenetic modifications such as DNA methylation may explain some of the missing heritability and could potentially inform biological knowledge of endocrine disease mechanisms involved in development of later life disease. Using data from the Avon Longitudinal Study of Parents and Children (ALSPAC), we explored cross-sectional associations of SHBG, total testosterone and bioavailable testosterone in childhood (males only) and adolescence (both males and females) with genome-wide DNA methylation. We also report associations of a *SHBG* polymorphism (rs12150660) with DNA methylation, which leads to differential levels of SHBG in carriers, as a genetic proxy of circulating SHBG levels.

**Results:**

We identified several novel sites and genomic regions where levels of SHBG, total testosterone, and bioavailable testosterone were associated with DNA methylation, including one region associated with total testosterone in males (annotated to the *KLHL31* gene) in both childhood and adolescence and a second region associated with bioavailable testosterone (annotated to the *CMYA5* gene) at both time-points. We also identified one region where both SHBG and bioavailable testosterone in males in childhood (annotated to the *ZNF718* gene) was associated with DNA methylation.

**Conclusion:**

Our findings have important implications in the understanding of the biological processes of SHBG and testosterone, with the potential for future work to determine the molecular mechanisms that could underpin these associations.

**Electronic supplementary material:**

The online version of this article (10.1186/s12863-018-0703-y) contains supplementary material, which is available to authorized users.

## Background

Epigenetic modifications such as DNA methylation can alter gene expression without changing underlying DNA sequences. Aberrant DNA methylation changes are known to be involved in causing disease, the most well-known examples being Rett syndrome [1] and cancer [2]. Differential DNA methylation has also been shown to be involved in common diseases such as type 2 diabetes [3]. There is much interest in such associations due to the potential to explain ‘missing’ heritability and inform biological knowledge of disease mechanisms that may lead to successful preventative or therapeutic interventions through drug development [4, 5].

Levels of sex hormone-binding globulin (SHBG) and testosterone have been previously associated with various diseases such as metabolic syndrome [6], type 2 diabetes [7, 8] and hormone-dependent cancers [9–11]. Response to androgens such as testosterone has been indicated to have an important role in prostate cancer progression [12]. Variation in SHBG and testosterone has been shown to be highly heritable [13–15] and previous genome-wide association studies have identified common variants strongly associated with both testosterone and SHBG [16, 17]. Although these variants explain a large portion of the heritability of testosterone and SHBG, there remains a large amount of unexplained heritability in both traits. Differential DNA methylation may further serve as a marker of future disease risk or may even mediate the effect of sex hormones on endocrine disease onset and progression.

The DNA-binding domains of androgen and estrogen receptors, like almost all nuclear receptors, have been shown to induce chromatin remodeling activity (conversion of euchromatin to inactive heterochromatin and vice-versa) [18] indicating a potentially direct role of sex hormones in epigenetic processes. Sex hormones are also thought to be involved in DNA methylation processes underpinning sexual differentiation, with evidence from animal models indicating that masculinizing genes in the fetal brain are subject to regulation by DNA methyltransferases [19]. In human populations, on an epigenome-wide level, previous studies have reported conflicting results regarding associations of sex hormones with DNA methylation and have been limited to assessing average methylation across the genome or in genomic repeats [20, 21]. Site-specific DNA methylation changes have not been assessed and therefore little can be concluded as to the effect of sex hormones on specific genes or biological pathways.

In this study, we explore associations of SHBG and testosterone with epigenome-wide DNA methylation in both childhood (for males) and adolescence (for males and females) that may a) explain some of the missing heritability of sex hormone levels and b) mediate risk of disease in later-life. We use complementary observational and genetic epidemiology approaches to strengthen our conclusions.

## Methods

### Data

#### Study population

The Avon Longitudinal Study of Parents and Children (ALSPAC) recruited 14,541 pregnant women resident in the former county of Avon, UK with expected dates of delivery 1st April 1991 to 31st December 1992. There were 14,541 initial pregnancies, for which the mother enrolled in the ALSPAC study and had either returned at least one questionnaire or attended a “Children in Focus” clinic by the 19th July 1999. Of the initial pregnancies, there were a total 14,062 live births and 13,988 children were alive at 1 year of age. The cohort profile paper describes the phases of enrolment in more detail [22]. A searchable data dictionary on the study website contains details of the data http://www.bris.ac.uk/alspac/researchers/data-access/data-dictionary/. All ALSPAC participants provided written informed consent. The ALSPAC Law and Ethics Committee and the local Research Ethics Committees (Bristol and Weston Health Authority: E1808, Southmead Health Authority: 49/89 and the Frenchay Health Authority: 90/8) granted initial ethical approval for the study, in accordance with the guidelines of the Declaration of Helsinki. Subsequent follow-up data collection was granted ethical approval from ethics committees as specified in Additional file 1.

### DNA methylation data

DNA methylation data was available for approximately 1000 mother-child pairs under ARIES, the Accessible Resource for Integrated Epigenomics Studies [21]. DNA methylation was assayed using the Illumina Infinium HumanMethylation 450 k BeadChip platform which measures methylation status of over 480,000 CpG sites across the genome. DNA methylation measures assayed at approximately 7.5 years (childhood) and 16.5 years (adolescence) were used in all analyses. Pre-processing of peripheral blood samples was performed as previously described [23]. A complete description of the DNA methylation data can be found in Additional file 1.

### Hormone data

#### Males

SHBG and total testosterone were measured using enzyme-linked immunosorbent assays in a subset of 513 male ALSPAC participants at 9.9 and 17.8 years of age. The quantification methods and assay standardisations used on male samples has been previously described [24]. Male measures of total testosterone were standardized by time-of-venipuncture (since testosterone displays a circadian rhythm) by using multilevel modelling to predict testosterone at a standard time of day, as described previously [24]. Measures of bioavailable testosterone were derived from measures of total testosterone (not corrected for time-of-venipuncture or exact age) and SHBG as previously described [24]. A complete description of the male hormone assays is available in Additional file 1.

#### Females

For the female samples, one SHBG and one total testosterone measure was made on serum samples obtained from females at 15.5 years. Assays in females were conducted at a different time and in a different way to those in males. Standardization by time-of-venipuncture was not performed on samples from females. A complete description is available in Additional file 1.

### Genetic data

Genetic data for the ALSPAC children were generated by Sample Logistics and Genotyping Facilities at the Wellcome Trust Sanger Institute and LabCorp (Laboratory Corporation of America) with support from 23andMe using the Illumina Human Hap 550-quad and the Illumina GenomeStudio calling algorithm. Details of genetic data QC can be found in Additional file 1.

### Statistical analyses

#### EWAS

Multiple linear regression was used to model methylation as the outcome and a measure of either SHBG or testosterone as the exposure. All measures of SHBG and testosterone were standardized to the mean (z-scored) to enable comparisons across time-points. Measures of SHBG or testosterone at 9.9 years were paired with DNA methylation at 7.5 years, and measures at 17.8 years were paired with DNA methylation at 16.5 years. Measures of SHBG and testosterone were therefore prospective (measured after the DNA methylation). However, in all models SHBG and testosterone were considered the exposures and DNA methylation the outcome. Results were corrected for multiple testing by controlling for the expected proportion of false-positives (FDR-adjusted *p*-value< 0.05). All analyses were stratified by sex, with males and females in separate models and all models were adjusted for batch effects and residual/unknown confounding by use of maximum 10 surrogate variables (SVs) using the sva R package [25]. Additionally, models were adjusted for maternal smoking during pregnancy, maternal age, parity, maternal education and estimated cell-counts derived using a reference dataset [26] and the Houseman method [27]. EWAS models were run in R version 3.2.2 using the CpGassoc package [28]. Results of fully adjusted models that include all the above-mentioned confounders are those presented in the results. Descriptions of the EWAS covariates can be found in Additional file 1.

#### EWAS of genotype

Observational associations between hormones measured from peripheral blood and DNA methylation are subject to confounding and reverse causation. Common genetic variants, such as SNPs, however, are randomised at birth and are therefore not subject to the same confounding structures. Therefore, we explored associations of DNA methylation with the rs12150660 polymorphism previously found to be associated with SHBG (*p*  =  2 × 10^− 106^) [16]. Up to 7.8% of the variation in SHBG in males and 3.3% of the variation in females is estimated to be due to the rs12150660 variant [16]. The polymorphism is in strong linkage disequilibrium (LD) with a nearby pentanucleotide repeat that is thought to directly affect SHBG expression levels [29]. A sex-stratified EWAS of the variant was performed, as a proxy for SHBG, in both childhood and adolescence, as we hypothesized that the effects of the polymorphisms would be different between males and females and would also differ prepubertally vs postpubertally. We adjusted for batch effects (by use of max 10 SVs), estimated cell counts and participants age but not for maternal confounders as we considered the variant independent of this type of maternal confounding. We also excluded CpGs on chromosome 17, the same chromosome as the variant since associations observed with CpGs on the same chromosome as the genetic variant are likely driven by LD.

#### DMR analysis

Differentially methylated regions (DMRs) were determined using comb-p [30]. Briefly, comb-p is a statistical software package that combines adjacent *p*-values based on calculations of their auto-correlation and assigns significance to regional enrichment after adjustments for multiple testing. It calculates adjusted *p*-values for each probe that accounts for the local correlation of that probe with its neighbouring probes. Probe-level *p*-values are then adjusted by using the Benjamini-Hochberg procedure, resulting in multiple testing corrected *p*-values for probes that are independent of their neighbouring CpGs. Next, Comb-p finds regions of differentially methylated regions and calculates *p*-values for the regions based on correlation (Stouffer-Liptak-Kechris (SLK) *p*-value). DMR *p*-values are finally adjusted using the Šidák correction based on the size of the region and number of possible regions of that size. Because comb-p combines *p*-values and does not consider effect sizes of individual CpGs, we additionally removed DMRs if more than half of the CpGs comprising that DMR had a regression coefficient with an absolute effect size of less than 0.01 (a 1% change in methylation per SD increase in exposure). DMR analysis was performed on all EWAS model results. A window size of 500 bases was used with the minimum number of significant probes required to start a DMR set to 2. We also excluded DMRs if they were comprised of only 2 CpGs with one of the two CpGs having an effect in the reverse direction to the other. Šidák corrected *p*-values < 0.05 were considered statistically significant.

#### Sensitivity analyses

Since only measures of total testosterone in males were standardized for time-of-venipuncture, we performed a sensitivity analysis adjusting for time-of-venipuncture as a covariate in the EWAS of bioavailable testosterone in order to determine the effects of circadian rhythm on the results. In the first of two sensitivity analyses, time-of-venipuncture was converted to a continuous measure by dividing the minutes of the hour in to 100ths and adding them to the hour. However, as the effects of time-of-venipuncture are unlikely to be linear, we performed a second sensitivity analysis where we adjusted for time as a categorical measure. We split time of day in to four approximately equal categories of 2-h increments; before (or at) 1000 h, 1000–1200 h, 1200–0200 h, and any time after 0200 h. We compared results of the EWAS sensitivity analyses with the main results.

## Results

### Sample characteristics

The distributions of SHBG, total testosterone, and bioavailable testosterone can be seen in Additional file 1: Figures S1, S2 and S3 respectively. Distributions of confounder data can be seen in Table 1. Females with data on SHBG and total testosterone in adolescence were more likely to have a mother of low education level (O-levels or lower) than males at the adolescent time-points.

**Table 1:** Descriptive statistics of the different EWAS time-points with distributions of covariates

Age^a^ (months) [sd]	N	Sex	N Parity (nulliparous) [%]	N Maternal smoking (never) [%]	Maternal age (years) [sd]	N Maternal education (O-levels or lower) [%]
SHBG						
117.8 [2.7]	113	Males	54 [47.8]	100 [88.5]	30.5 [4.2]	45 [39.8]
184.7 [3.4]	359	Females	179 [49.9]	314 [87.5]	29.6 [4.2]	180 [50.1]
211.8 [3.9]	114	Males	55 [48.2]	103 [90.4]	30.9 [4.4]	41 [36.0]
Total testosterone^b^						
117.8 [2.7]	113	Males	54 [47.8]	100 [88.5]	30.5 [4.2]	45 [39.8]
184.8 [3.4]	375	Females	182 [48.5]	329 [87.7]	29.6 [4.2]	191 [50.9]
211.8 [3.9]	113	Males	55 [48.7]	102 [90.3]	30.9 [4.4]	40 [35.4]
Bioavailable testosterone						
117.8 [2.7]	113	Males	54 [47.8]	100 [88.5]	30.5 [4.2]	45 [39.8]
211.8 [3.9]	113	Males	55 [48.7]	102 [90.3]	30.9 [4.4]	40 [35.4]

^a^Age at time of hormone measurement

^b^Total testosterone standardized for exact age and time-of-venipuncture in males but not in females

### EWAS

**Fig. 1:**
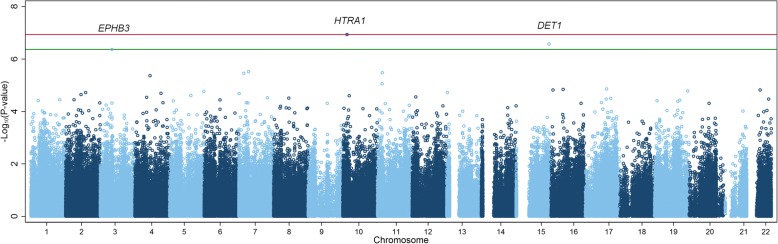
Manhattan plot of SHBG in females (adolescence)

There were no single-site associations for either SHBG, total testosterone, or bioavailable testosterone in any of the EWAS in males. None of the CpG sites survived FDR correction for multiple testing (FDR adjusted *p*-value< 0.05). There were 3 CpGs associated with SHBG in females in adolescence. These CpGs mapped to *HTRA1* (cg01962937, *p* = 1.18E-07), *DET1* (cg08724901, *p* = 2.70E-07) and *EPHB3* (cg06577604, *p* = 4.36E-07) genes (Fig. 1). However, effect sizes were small for all 3 CpGs (Additional file 2: Table S6). There was little evidence of inflation in adjusted models of total testosterone or bioavailable testosterone, assessed using the lambda (λ) inflation statistic and QQ-plots (Additional file 1: Figures S4, S5 and S6). Similarly, for SHBG there was little evidence of inflation, except for the female time-point at 15.5 years, where λ = 1.17 and some inflation was visible in the QQ-plot.

In the EWAS of SHBG genotype, the number of individuals with available data was *N* = 409 for adolescent males, *N* = 433 for adolescent females, *N* = 420 for male children and *N* = 421 for female children. There were no single site associations in any of the models for females or males. There was little evidence for inflation in any of the models, except for adolescent females where λ = 1.13 (Additional file 1: Figure S7).

Full results of each EWAS model are available in Additional file 2.

### DMRs

There was 1 DMR associated with SHBG in childhood in males, annotated to the *ZNF718* gene (Table 2). There were no DMRs associated with SHBG in adolescence in males. In females, there were 2 DMRs associated with SHBG in adolescence in females. There were no DMRs associated with testosterone in adolescence in females (Table 2).

**Table 2:** Differentially methylated regions (DMRs) associated with SHBG, total testosterone and bioavailable testosterone

	Differentially methylated region (DMR)	N CpGs	Ŝidak *p*-value	Gene	Range^a^
SHBG					
Adolescence					
Females	Chr8:57350735–57351068	5	1.1E-04	*–*	0.014, 0.035
Females	Chr16:53407594–53407809	3	3.8E-03	*–*	0.011, 0.031
Childhood					
Males	Chr4:124232–124623	3	9.9E-04	*ZNF718*	0.0053, 0.018
Total testosterone					
Childhood					
Males	Chr3:126911727–126911954	4	8.4E-06	*–*	− 0.038, − 0.015
Males	Chr10:10337051–10337313	2	1.4E-04	*–*	−0.034, − 0.02
Males	Chr6:53530503–53530629	4	2.6E-04	*KLHL31*	−0.022, − 0.0091
Adolescence					
Males	Chr6:53530503–53530629	4	1.1E-05	*KLHL31*	−0.022, − 0.012
Males	Chr4:57547347–57547700	2	9.5E-04	*HOPX*	−0.035, − 0.027
Males	Chr15:93580022–93580328	4	1.3E-03	*–*	−0.055, − 0.013
Bioavailable testosterone					
Childhood					
Males	Chr4:124232–124694	4	9.3E-09	*ZNF718*	−0.019, − 0.0064
Males	Chr5:78985432–78985593	7	2.4E-04	*CMYA5*	−0.031, − 0.024
Males	Chr15:26874098–26874364	3	8.0E-03	*GABRB3*	−0.037, − 0.024
Adolescence					
Males	Chr5:78985432–78985901	8	6.5E-05	*CMYA5*	−0.044, − 0.022

^a^Range of effect sizes observed across CpGs comprising that DMR reported to 2 significant figures, where estimates are change in methylation beta per SD increase in SHBG or testosterone

**Fig. 2:**
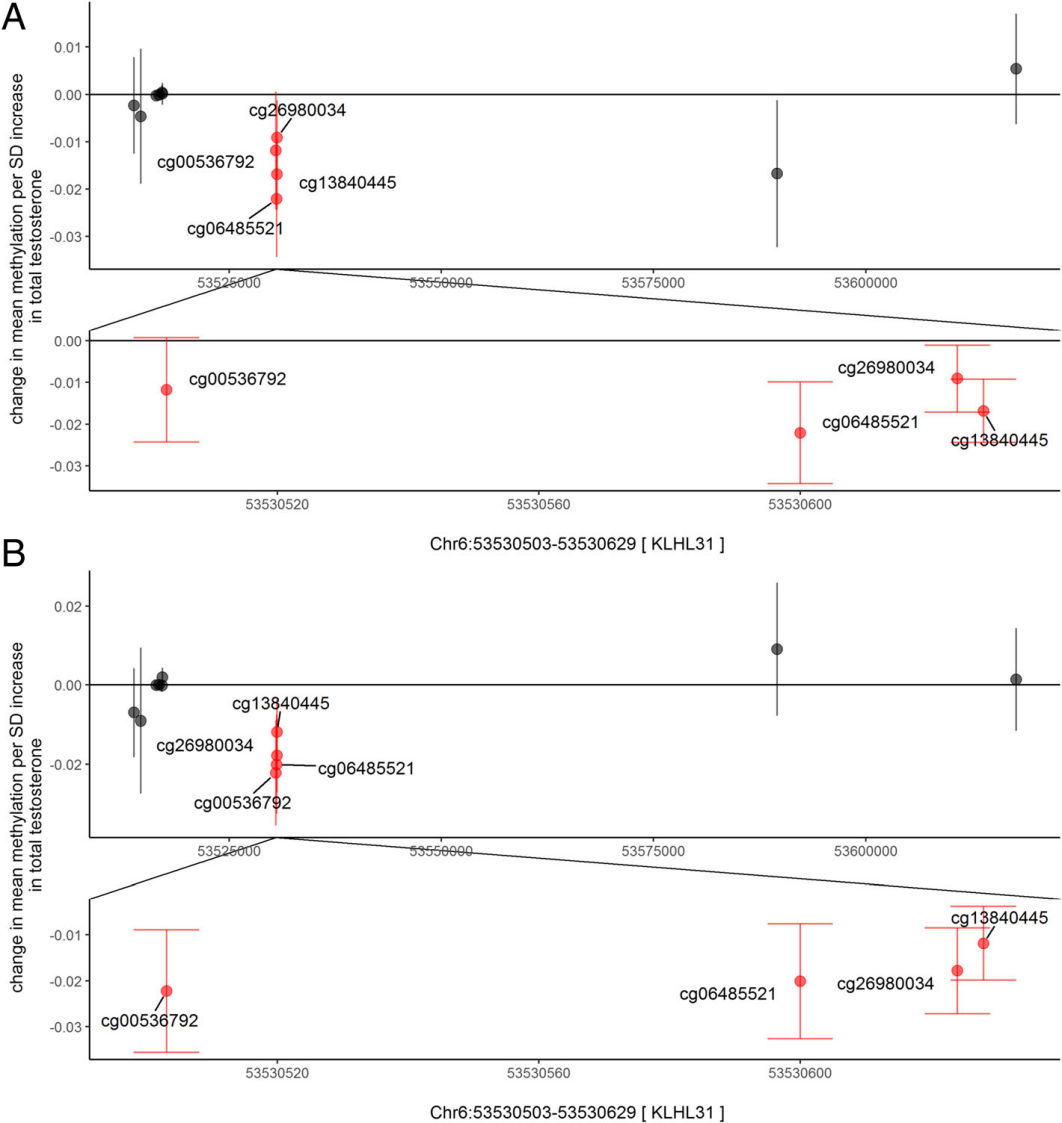
Comparison of Chr6[*KLHL31*] DMR associated with total testosterone in males in (**a**) childhood (at 7.5 years of age), (**b**) adolescence (at 16.5 years of age)

**Fig. 3:**
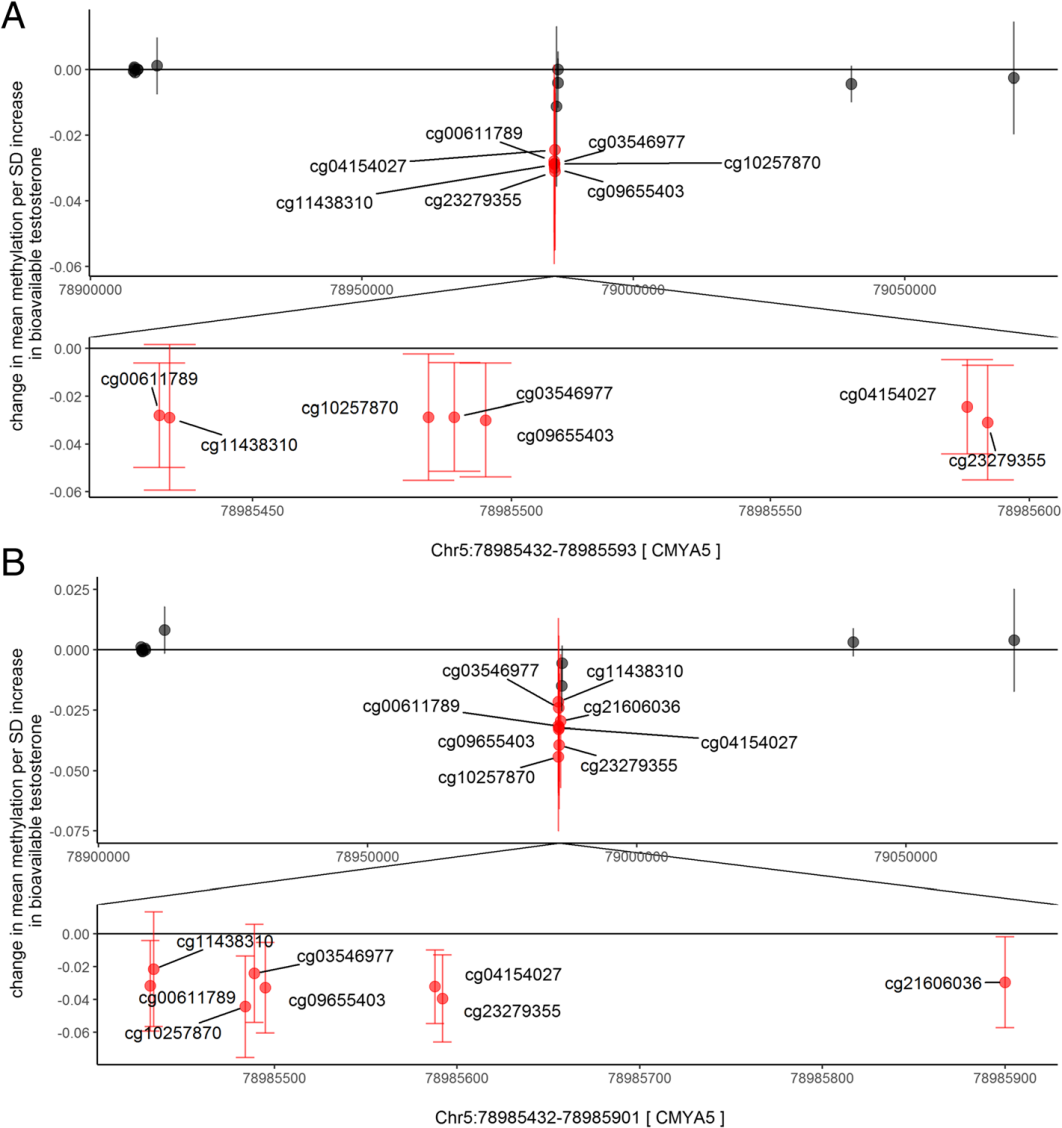
Comparison of Chr5[*CMYA5*] DMR associated with bioavailable testosterone in males in (**a**) childhood (at 7.5 years of age), (**b**) adolescence (at 16.5 years of age)

In males, there were 3 DMRs associated with total testosterone in childhood and 3 DMRs associated with total testosterone in adolescence (Šidák corrected *p*-value < 0.05), with one DMR in common between the two time-points on chromosome 6, annotated to the *KLHL31* gene (Fig. 2) where an increase in total testosterone was associated with a decrease in DNA methylation at that locus. There were 3 DMRs associated with bioavailable testosterone in childhood and 1 DMR associated with bioavailable testosterone in adolescence. There was one DMR in common between the two time-points on chromosome 5, annotated to the *CMYA5* gene (Fig. 3). where an increase in bioavailable testosterone was associated with a decrease in DNA methylation at that locus. There was no overlap between DMRs in males and females from the bioavailable testosterone models, the total testosterone models or the SHBG models.

There was one DMR in common between the models of SHBG and bioavailable testosterone in childhood in males annotated to the *ZNF718* gene on chromosome 4. The effect sizes were reversed between models with a maximum 0.018 increase in methylation beta per SD increase in SHBG, and a maximum 0.019 decrease in methylation beta per SD increase in bioavailable testosterone at CpGs comprising that DMR.

In the EWAS of SHBG genotype, there were 2 DMRs associated with rs12150660 in childhood for females annotated to the *FZD7* and *TMEM132D* genes. There was 1 DMR associated with rs12150660 in adolescent females annotated to the *ACY3* gene. The were no DMRs associated with the genotype in males in either childhood or adolescence (Table 3). None of the DMRs overlapped between models and there was no overlap between DMRs from the EWAS of genotype and those from the observational EWAS.

**Table 3:** DMRs associated with the rs12150660 *SHBG* polymorphism

	Differentially methylated region (DMR)	N CpGs	Ŝidak *p*-value	Gene	Range^a^
SHBG genotype					
Childhood					
Females	Chr2:202901045–202901471	5	1.9E-03	*FZD7*	0.026, 0.046
Females	Chr12:130060028–130060081	2	9.9E-03	*TMEM132D*	0.009, 0.018
Adolescence					
Females	Chr11:67418045–67418406	10	7.8E-06	*ACY3;SMAD3*	*−0.024, −0.0036*

^a^Range of effect sizes observed across CpGs comprising that DMR, where estimates are change in methylation beta per copy of rs12150660 variant allele

Full results for both the genetic and the observational DMR analysis can be seen in Additional file 3.

### Sensitivity analyses

In a series of sensitivity analyses exploring the effects of circadian rhythm of testosterone on EWAS results we determined that time-of-venipuncture did not have a substantial effect on detected associations. Adjusting EWAS models of bioavailable testosterone in males in adolescence for time-of-venipuncture as a continuous measure or as a categorical measure did not attenuate the associations of the detected DMRs. Similarly, for bioavailable testosterone in childhood all 3 DMRs remained associated with the trait (Ŝidak *p*-value< 0.05) following adjustment. We did not perform the same sensitivity analysis for testosterone in females as we did not detect any associations in the unadjusted models and inflation was low (λ = 0.90) indicating little unexplained confounding.

## Discussion

In this epigenome-wide association study, the first to explore cross-sectional associations of SHBG and testosterone with genome-wide DNA methylation, we identified several single-sites and regions associated with SHBG and testosterone. One region annotated to the *CMYA5* gene in males that appeared to be differentially methylated in both childhood and adolescence, with an increase in bioavailable testosterone associated with a decrease in methylation. We also identified one region mapping to the *KLHL31* gene, with an increase in total testosterone in males in both childhood and adolescence associated with a decrease in DNA methylation. The fact that DNA methylation associations at these two regions persists over time may indicate that the sites are influenced by underlying genetics. However, neither of the two genes have ever been identified in previous GWAS of SHBG or testosterone.

We considered the study underpowered to attempt a longitudinal analysis at a genome-wide level using the current dataset, as modelling change over time would most probably require far larger sample sizes. Attempting to identify methylation signals in childhood that predict later hormone levels would similarly require far larger numbers.

Notably, we did not detect any overlap between the DMRs from the EWAS of SHBG genotype (rs12150660 variant) and those from the observational EWAS. This may indicate that associations in the observational EWAS are due to reverse causation (i.e. DNA methylation causing changes in SHBG or testosterone) or due to unmeasured confounding. Whereas the genetic variant may be expected to proxy average lifetime exposure to SHBG up until the time of DNA methylation measurement (that includes in utero effects), SHBG measured from peripheral blood may capture a more temporal effect of SHBG on DNA methylation, potentially explaining the lack of overlap. There was also no overlap between the two sexes, indicating that SHBG and testosterone may have sex-specific effects on DNA methylation.

We assessed the effects of circadian rhythm on bioavailable testosterone in males and found little evidence of any confounding effects of time-of-day. Whether SHBG is also subject to diurnal variation has not been precisely determined [31]. In our sample, we assessed the effects of diurnal variation on the testosterone-DNA methylation associations and found little effect or attenuation of signal following adjustments. We therefore considered any potential effect of diurnal variation of SHBG (where previous evidence of variation is less clear) to be relatively minor.

We observed consistently high inflation of *p*-values, assessed using the inflation factor lambda, in the EWAS of SHBG in females when compared to other models. In both the EWAS of SHBG genotype and the EWAS of serum SHBG, inflation lambda was greater than 1.13 indicating residual confounding or unexplained variation. Given the excess significance in models of SHBG in females, the single-site associations observed between DNA methylation at 3 CpGs and SHBG in that model may represent false positives. However, since high inflation was not observed in other models, this observation may be consistent with SHBG having multiple small effects on DNA methylation throughout the epigenome in post-pubertal females.

### Functional significance

If SHBG and testosterone do causally influence blood DNA methylation in the general population under normal physiological conditions, perturbations in DNA methylation and expression levels of the annotated genes may indicate hypogonadism or hypergonadism, as well as other diseases such as type-2 diabetes that SHBG has been previously causally linked to [7]. Disruptions to these DNA methylation patterns has also been suggested to be tied to the development of other endocrine diseases [32] and may also mark onset of diseases such as prostate cancer, in which sex hormones such as testosterone have been shown to be involved [33]. Further investigation of the biological mechanism behind these associations may provide important mechanistic understanding of the regulatory processes of SHBG and testosterone. Associations may also have important prognostic value in the progression of diseases such as type-2 diabetes and prostate cancer.

We did not identify enough CpGs between the single-site analysis and the analysis of DMRs to provide sufficient statistical power to perform a functional analysis. We instead performed a literature search of the two genes, *CMYA5* and *KLHL31*, annotated to DMRs intersecting the child and adolescence models. Both genes were found to be involved in skeletal and/or cardiac muscle regeneration. The *CMYA5* gene encodes myospryn, a protein expressed predominantly in cardiac/skeletal muscle, with a suggested role in muscle regeneration [34, 35]. Previous disease associated with *CMYA5* include Duchenne muscular dystrophy [36, 37] and schizophrenia [38]. The *KLHL31* gene encodes kelch-like protein 31, a protein involved in skeletal muscle myogenesis [39]. Polymorphisms in *KLHL31* have previously been associated with loin muscle area in porcine models [40]. This hypothetical biological pathway requires further interrogation, but would be of interest due to the anabolic insights it might uncover and therefore potential mechanisms that might be relevant to muscle wastage (cachexia).

### Strengths and limitations

One of the major strengths of our study is the ability to compare cross-sectional time-points of DNA methylation and hormone exposures, making pairwise comparisons of EWAS results between different measures in both sexes. We have also strengthened our conclusions by combining observational evidence from EWAS of serum/plasma SHBG and testosterone with that using SHBG genotype.

We were however restricted by a small sample size, particularly in EWAS of male hormone measures. In female models, sample size was larger but EWAS may still have suffered from low power, particularly if SHBG and testosterone have only small effects on the epigenome. Similarly, for the EWAS of SHBG genotype where the genetic variant explains only a fraction of variation in circulating SHBG, the analyses may have been underpowered to detect small effects. We have not attempted to replicate our results in a different collection of data as we were restricted by the scarcity of cohorts with both DNA methylation and sex hormone data. Future studies should focus on attempting to confirm the current results in further datasets.

We were further limited by opportunistic availability of the data, with total testosterone available only in standardized (for time-of-venipuncture and exact age) form and bioavailable testosterone available only in raw unadjusted form. Furthermore, female measurements were assayed from serum at a different time and using different methods to the male data which was assayed from plasma. Although we do not expect to see differences between serum and plasma (serum is simply plasma that has clotted), the different methods used reduce our ability for comparisons between the two sexes due to potential batch effects.

The male measures of SHBG and testosterone used in our analyses have been previously compared to published reference datasets, with results indicating that the values fall within the expected ranges [24]. However, 136 testosterone samples (26.5% of the total sample) from the childhood time-point were set to the lowest assay sensitivity cutoff as testosterone levels were below the assay sensitivity threshold, potentially resulting in bias towards the null. This is a common problem of assays of testosterone in prepubertal males, where serum/plasma testosterone levels are low. We were unable to assess the effects of maternal sex hormones on the observed associations. However, previous evidence has indicated that effects of maternal exposures on the offspring epigenome attenuate after the early pre-natal period [41] and are unlikely to impact our results substantially.

We could only assess associations for a fraction of sites in the epigenome since the coverage of the array used for measuring DNA methylation (HumanMethylation450 Beadchip array) is limited (70). Additionally, DNA methylation was measured in peripheral blood which may not be the most relevant tissue in which to assess the effects of sex hormones. Cell type proportions for the peripheral blood samples were estimated from the DNA methylation data using an external reference. This method of deriving cell counts has been previously validated (71) and is commonly used, however, derived cell counts but may be an imprecise estimation of true cell type proportions.

## Conclusion

We have identified several novel sites and genomic regions where levels of SHBG, total testosterone and bioavailable testosterone are associated with DNA methylation. We also report several associations of a polymorphism in the *SHBG* gene which leads to differential levels of SHBG in carriers with DNA methylation. Findings have important implications in the understanding of biological processes of SHBG and testosterone, with the potential for future work to determine the molecular mechanisms that underpin these associations that may be involved in endocrine disease processes.

## Additional files

Additional file1: Supplementary Methods of ALSPAC data description and Results **Table S1.** SHBG, total testosterone and bioavailable testosterone measures available in ALSPAC. Values presented for all ALSPAC participants regardless of DNA methylation data availability. **Figure S1.** Distributions of SHBG in males at 9.9, 17.8 years and in females at 15.5 years. Graphs include only individuals with available DNA methylation data in ARIES. **Figure S2.** Distributions of total testosterone in males at 9.9 and 17.8 years and in females at 15.5 years. Graphs include only individuals with available DNA methylation data in ARIES. **Figure S3.** Distributions of bioavailable testosterone in males at 9.9 and 17.8 years. Graphs include only individuals with available DNA methylation data in ARIES. **Figure S4.** QQ plots of SHBG EWAS at 9.9 and 17.8 years in males and 15.5 years in females. **Figure S5.** QQ plots of total testosterone EWAS at 9.9 and 17.8 years in males and 15.5 years in females. **Figure S6.** QQ plots of total testosterone EWAS at 9.9 and 17.8 years in males. **Figure S7.** QQ-plots of EWAS models of rs12150660 in childhood and adolescence, stratified by sex. (DOCX 472 kb)

Additional file 2: **Table S1.** EWAS results of total testosterone in childhood in males (*P* value< 0.005). **Table S2.** EWAS results of total testosterone in adolescence in males (*P* value< 0.005). **Table S3.** EWAS results of total testosterone in adolescence in females (*P* value< 0.005). **Table S4.** EWAS results of SHBG in childhood in males (*P* value< 0.005). **Table S5.** EWAS results of SHBG in adolescence in males (*P* value< 0.005). **Table S6.** EWAS results of SHBG in adolescence in females (*P* value< 0.005). **Table S7.** EWAS results of bioavailable testosterone in childhood in males (*P* value< 0.005). **Table S8.** EWAS results of bioavailable testosterone in adolescence in males (*P* value< 0.005). **Table S9.** EWAS results of the rs12150660 variant in childhood in males (*P* value < 0.005). **Table S10.** EWAS results of the rs12150660 variant in childhood in females (*P* value< 0.005). **Table S11.** EWAS results of the rs12150660 variant in adolescence in males (*P* value< 0.005). **Table S12.** EWAS results of the rs12150660 variant in adolescence in females (*P* value< 0.005). (XLSX 1551 kb)

Additional file 3: **Table S1.** DMR analysis of total testosterone in childhood in males. **Table S2.** DMR analysis of total testosterone in adolescence in males. **Table S3.** DMR analysis of SHBG in childhood in males. **Table S4.** DMR analysis of SHBG in adolescence in females. **Table S5.** DMR analysis of bioavailable testosterone in childhood in males. **Table S6.** DMR analysis of bioavailable testosterone in adolescence in males**. Table S7.** DMR analysis of the rs121S50660 polymorphism of the SHBG gene in childhood in females. **Table S8.** DMR analysis of the rs12150660 polymorphism of the SHBG gene in adolescence in females. (XLSX 18 kb)

## Abbreviations

ALSPAC: Avon Longitudinal Study of Parents and Children
ARIES: Accessible Resource for Integrated Epigenomics Studies
CpG: Cytosine-phosphate-Guanine
DMR: Differentially methylated region
EWAS: Epigenome wide association study
FDR: False discovery rate
GWAS: Genome wide association study
LD: Linkage disequilibrium
QC: Quality control
QQ: Quantile quantile
SD: Standard deviation
SHBG: Sex hormone-binding globulin
SNP: Single nucleotide polymorphism
SV: Surrogate variables
