# Supplementary material for: Associations of sex hormone-binding globulin and testosterone with genome-wide DNA methylation
Source: BMC Genet. 2018 Dec 14;19:113. doi: 10.1186/s12863-018-0703-y (PMC6295101; doi:10.1186/s12863-018-0703-y)
Supplement: Supplementary file 1 — Supplementary Methods of ALSPAC data description and Results Table S1. SHBG, total testosterone and bioavailable testosterone measures available in ALSPAC. Values presented for all ALSPAC participants regardless of DNA methylation data availability. Figure S1. Distributions of SHBG in males at 9.9, 17.8 years and in females at 15.5 years. Graphs include only individuals with available DNA methylation data in ARIES. Figure S2. Distributions of total testosterone in males at 9.9 and 17.8 years and in females at 15.5 years. Graphs include only individuals with available DNA methylation data in ARIES. Figure S3. Distributions of bioavailable testosterone in males at 9.9 and 17.8 years. Graphs include only individuals with available DNA methylation data in ARIES. Figure S4. QQ plots of SHBG EWAS at 9.9 and 17.8 years in males and 15.5 years in females. Figure S5. QQ plots of total testosterone EWAS at 9.9 and 17.8 years in males and 15.5 years in females. Figure S6. QQ plots of total testosterone EWAS at 9.9 and 17.8 years in males. Figure S7. QQ-plots of EWAS models of rs12150660 in childhood and adolescence, stratified by sex. (DOCX 472 kb) [file 12863_2018_703_MOESM1_ESM.docx]

# Supplementary Methods

# ALSPAC

## Design and study population

ALSPAC is a large, prospective cohort study based in the South West of England^(1)^. 14,541 pregnant women resident in Avon, UK with expected dates of delivery 1st April 1991 to 31st December 1992 were recruited and detailed information has been collected on these women and their offspring at regular intervals. The study website contains details of all the data that is available through a fully searchable data dictionary (http://www.bris.ac.uk/alspac/researchers/data-access/data-dictionary/).

Written informed consent has been obtained for all ALSPAC participants. The ALSPAC Law and Ethics Committee and the Local Research Ethics Committees (Bristol and Weston Health Authority: E1808, Southmead Health Authority: 49/89 and the Frenchay Health Authority: 90/8) granted initial ethical approval for the study, in accordance with the guidelines of the Declaration of Helsinki. Subsequent follow-up data collection was granted ethical approval from various ethics committees. For the 7 year clinic approval was granted from United Bristol Healthcare Trust: E4168, Southmead Health Services: 67/98 and Frenchay Healthcare Trust: 98/52. For the 15 year clinic, Central & South Bristol Research Ethics Committee (UBHT): 06/Q2006/53. The 17 year clinic was granted approval from North Somerset & South Bristol Research Ethics Committee: 08/H0106/9. A complete list of ethics approvals is listed on the study website: <http://www.bristol.ac.uk/alspac/researchers/research-ethics/>

## DNA Methylation measurements

Peripheral blood samples (whole blood or buffy coats) were collected according to standard procedures, spun and frozen at -80˚C. DNA methylation analysis and data pre-processing were performed at the University of Bristol as part of the ARIES project^(2)^ (ariesepigenomics.org.uk). Following extraction, DNA was bisulfite converted using the Zymo EZ DNA MethylationTM kit (Zymo, Irvine, CA). Following conversion, the genome-wide methylation status of over 485,000 CpG sites was measured using the Illumina Infinium® HumanMethylation450k BeadChip assay according to the standard protocol. The arrays were scanned using an Illumina iScan and initial quality review was assessed using GenomeStudio (version 2011.1). The level of methylation is expressed as a “Beta” value (β-value), ranging from 0 (no cytosine methylation) to 1 (complete cytosine methylation). Samples from all time-points in ARIES were distributed across slides using a semi-random approach (sampling criteria were in place to ensure that all time-points were represented on each array) to minimize the possibility of confounding by batch effects. Samples failing quality control (average probe detection p-value ≥ 0.01) were repeated. As an additional quality control step genotype probes on the HumanMethylation450k were compared between samples from the same individual and against SNP-chip data to identify and remove any sample mismatches. Data were pre-processed in R (version 3.0.1) with the WateRmelon package according to the subset quantile normalization approach described by Touleimat & Tost in an attempt to reduce the non-biological differences between probes.

Sites on sex chromosomes were excluded to reduce complexity due to sex-specific differences and X-chromosome inactivation by DNA methylation in females. Probes identified by Naeem et al.^(3)^ that map to multiple genomic locations, contain known repeat regions, INDELs or SNPs, or are affected by other unknown factors were excluded. Outliers were also removed from the methylation data using Tukey’s method^(4)^ of outlier removal as certain extreme methylation values caused by technical artefacts or rare genetic variants skewed the analysis. Potential outliers in the methylation data were removed if their value was less than the lower quartile minus three times the interquartile range or more than the upper quartile plus three times the interquartile range. Finally, probes showing a detection P-value >0.05 for >5% samples were excluded. This left 285,929 and 285,656 probes for analysis in peripheral blood in childhood and peripheral blood in adolescence, respectively.

## Covariates

Maternal age at delivery was derived from the mother’s report of her own and her baby’s dates of birth. Maternal social class (proxied by maternal education level) was classified for this study as “University” or “A-level” or “O-level or lower” based on questionnaires completed by the mothers. Maternal smoking behaviour was assessed during pregnancy via questionnaire and categorised for this study as 1) never smoking during pregnancy, 2) temporary smoking during pregnancy (if mothers only smoked in the first trimester), and 3) smoking throughout pregnancy. Parity was abstracted from maternal questionnaires and was categorised as 1) nulliparous or 2) multiple pregnancies. Ten surrogate variables^(5)^ were generated and included in models to adjust for technical batch.

## Cell type correction

Cell type correction was applied using the reference-based Houseman method^(6)^ in the minfi package^(7)^ in R. This method estimates the relative proportions of seven white blood cell subtypes (CD4+ T-lymphocytes, CD8+ T-lymphocytes, NK (natural killer) cells, B-lymphocytes, monocytes, eosinophils and neutrophils), based on a standard reference population^(8)^.

## Genetic data

Genetic data for the ALSPAC children were generated by Sample Logistics and Genotyping Facilities at the Wellcome Trust Sanger Institute and LabCorp (Laboratory Corporation of America) with support from 23andMe using the Illumina Human Hap 550-quad and the Illumina GenomeStudio calling algorithm. SNPs with more than 5% of missingness, a Hardy-Weinberg-Equilibrium p-value lower than 10^−6^, a minor allele frequency of less than 1%, indeterminate X chromosome heterozygosity or extreme autosomal heterozygosity were removed. SNP imputation was carried out against the 1000 Genomes Project database (www.1000genomes.org).

## Hormone measurements

### Males

Measurements of SHBG and total testosterone were available in a subset of ALSPAC participants. SHBG and total testosterone were measured in peripheral blood (lithium heparin plasma) in a subset of 513 males at 9.9 and 17.8 years. The quantification methods and assay standardisations used on male samples has been previously described ^(9)^. Briefly, enzyme-linked immunosorbent assays were used to measure plasma concentrations of SHBG and testosterone in blood samples, using commercially available kits. Assays had lower limits of sensitivity of 0.77 nmol/L (SHBG) and 0.28 nmol/L (testosterone). For testosterone, samples that yielded undetectable hormone values or values below the lower limit of sensitivity for the assay (n =136 from the 9-year visit) were set to the lower bound value of the detectable range for the assay (0.28 nmol/L).

In order to standardise male total testosterone measures by time of venipuncture (since testosterone displays a circadian rhythm) multilevel modelling was used to predict testosterone at a standard time of day, as described previously ^(9)^. Separate models were fit for each time-point as the effect of time of venipuncture varied by age. Age was included as a continuous variable in these models. The time-corrected values of total testosterone were those used in downstream analyses.

Measures of bioavailable testosterone were derived from measures of total testosterone (not corrected for time of venipuncture or exact age) and SHBG as previously described ^(9)^. Briefly, the equation “Total testosterone= Free testosterone + SHBG-bound testosterone + albumin-bound testosterone” was used, reliant on measures of SHBG assayed from the same samples and estimated concentrations of albumin-bound testosterone from a reference sample.

### Females

For the female samples, one SHBG and one total testosterone measure was made on serum samples obtained from females at age 15.5 years. Assays in females were conducted at a different time and in a different way to those in males. About half of the blood samples were drawn between 0800 and 0900 hours and the other half between 1200 and 1500 hours. SHBG was measured using a Cobas Auto Analyzer (Roche Diagnostic, West Sussex, UK) and SHBG reagent using the manufacturer’s calibrators and quality control material. Total testosterone was measured using Agilent triple quadrupole 6410 liquid chromatography/mass spectrometry equipment with an electrospray ionization source operating in positive ion mode (Agilent Technologies, Wilmington, DE, USA). Multiple reaction monitoring was used to quantify total testosterone by using trideuterated testosterone (d3t-testosterone), with the following transitions: m/z 289.2-97 and 289.2-109 for testosterone and 292.2-97 and 292.2-109 for d3t-testosterone. Measurements of SHBG and testosterone have been described previously^(10)^. Standardization by time of venipuncture was not performed on samples from females. For SHBG, measures exceeding the assay sensitivity threshold of 200 nmol/L were removed (N=6). We observed outliers in measures of testosterone in females and thus values were removed using Tukeys method of outlier removal ^(4)^. Values were removed if they were outside the range of ([25th percentile – 3 times the interquartile range) and (75th percentile + 3 times the interquartile range]).

# Supplementary Results

Table S1 SHBG, total testosterone and bioavailable testosterone measures available in ALSPAC. Values presented for all ALSPAC participants regardless of DNA methylation data availability.

| N | Mean | SD | Min | Max | ALSPAC clinic * | Age (years) | Sex |
| --- | --- | --- | --- | --- | --- | --- | --- |
| SHBG (nmol/L) | | | | | | | |
| 442 | 92.28 | 43.64 | 4.33 | 262.29 | F9 | 9.9 | Males |
| 428 | 26.25 | 12.37 | 3.35 | 140.09 | TF4 | 17.8 | Males |
| 1728 | 63.91 | 35.73 | 3.62 | 201.00 | TF3 | 15.5 | Females |
| Total testosterone (nmol/L) ^†^ | | | | | | | |
| 441 | 0.82 | 0.09 | 0.74 | 1.39 | F9 | 9.9 | Males |
| 413 | 16.50 | 2.65 | 8.68 | 28.59 | TF4 | 17.8 | Males |
| 1783 | 0.90 | 0.45 | 0.11 | 8.18 | TF3 | 15.5 | Females |
| Bioavailable testosterone (nmol/L) | | | | | | | |
| 441 | 0.17 | 0.07 | 0.06 | 0.64 | F9 | 9.9 | Males |
| 413 | 9.01 | 1.97 | 1.17 | 16.04 | TF4 | 17.8 | Males |

* *Where F9= Focus @ 9, TF3= Teen Focus 3, TF4=Teen Focus 4*

† *Total testosterone standardized for exact age and time of venipuncture in males but not in females.*


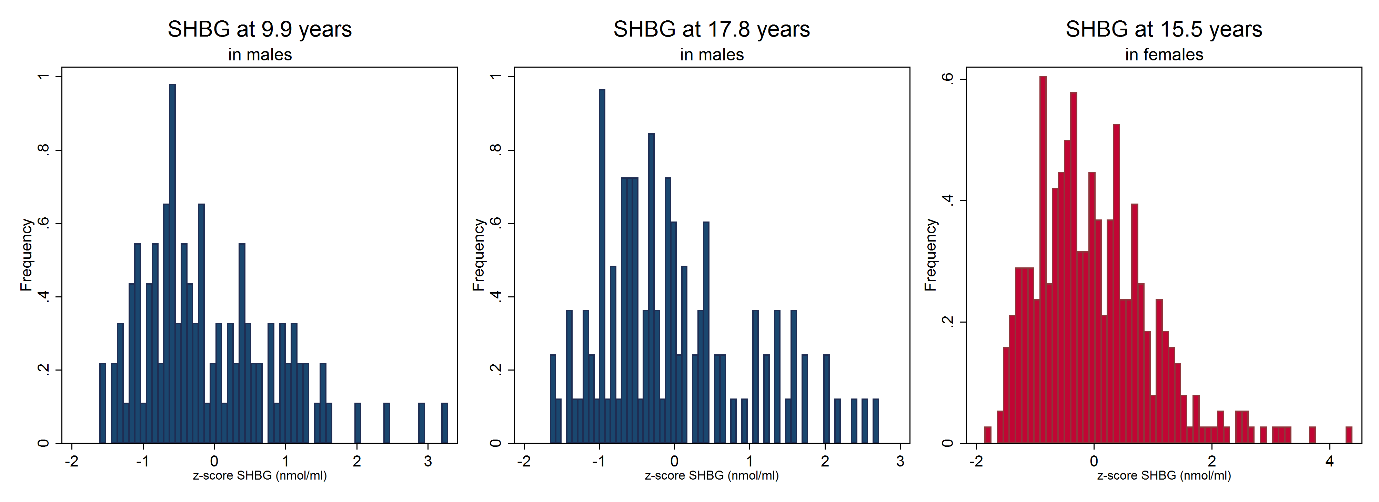


Figure S1 Distributions of SHBG in males at 9.9, 17.8 years and in females at 15.5 years. Graphs include only individuals with available DNA methylation data in ARIES.


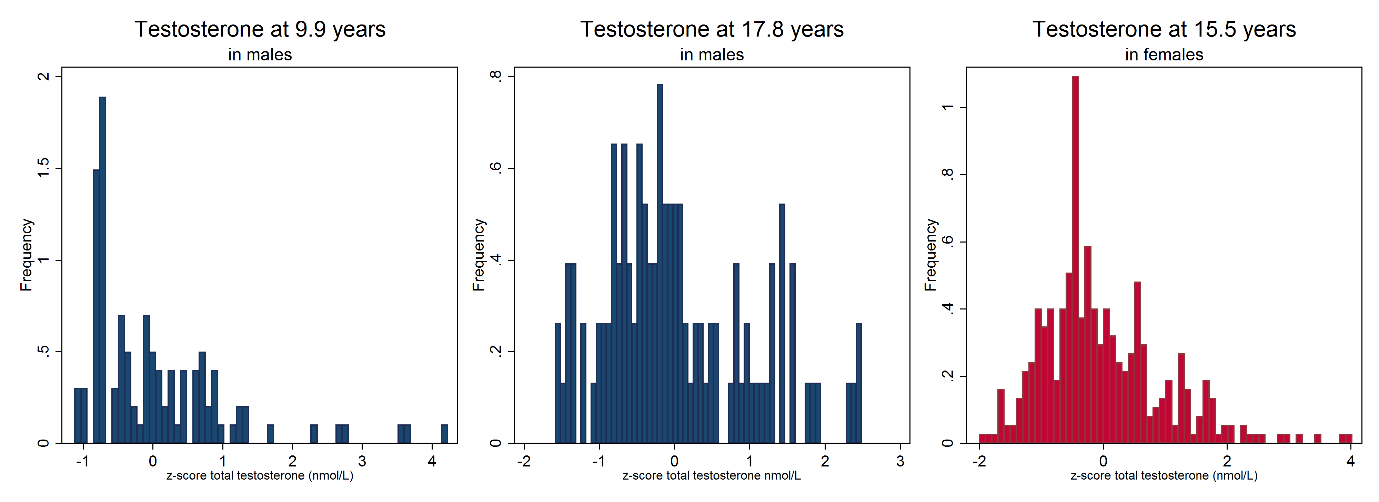


Figure S2 Distributions of total testosterone in males at 9.9 and 17.8 years and in females at 15.5 years. Graphs include only individuals with available DNA methylation data in ARIES.


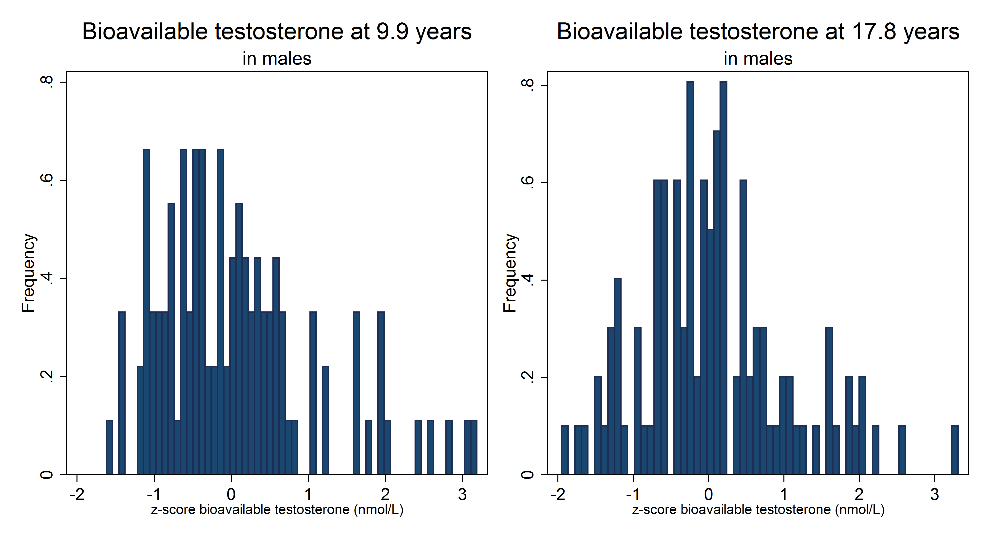


Figure S3 Distributions of bioavailable testosterone in males at 9.9 and 17.8 years. Graphs include only individuals with available DNA methylation data in ARIES.


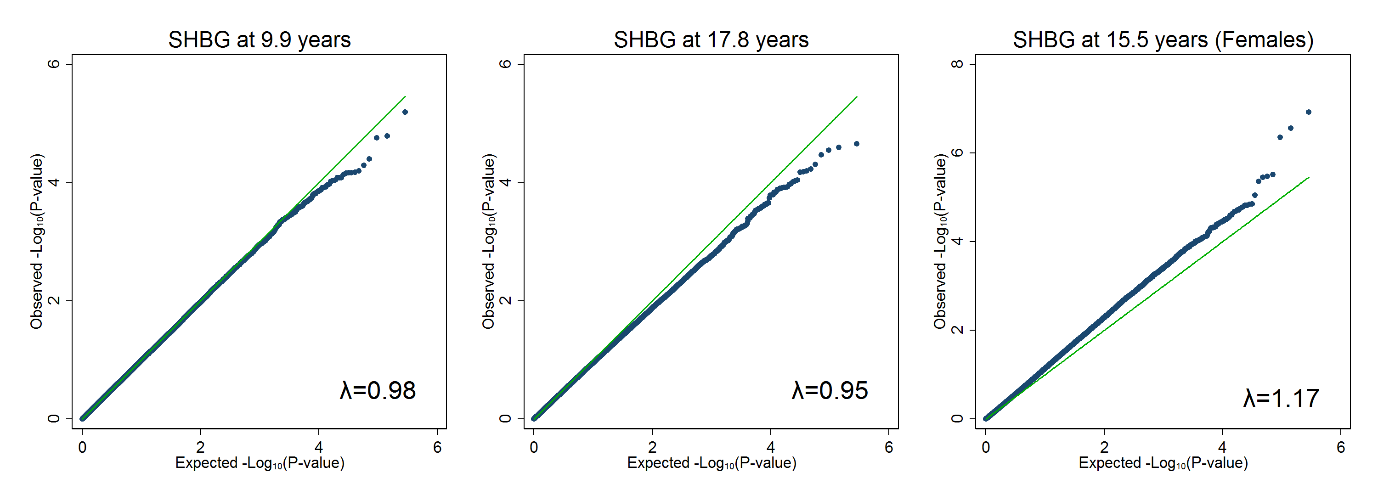


Figure S4 QQ plots of SHBG EWAS at 9.9 and 17.8 years in males and 15.5 years in females.

*
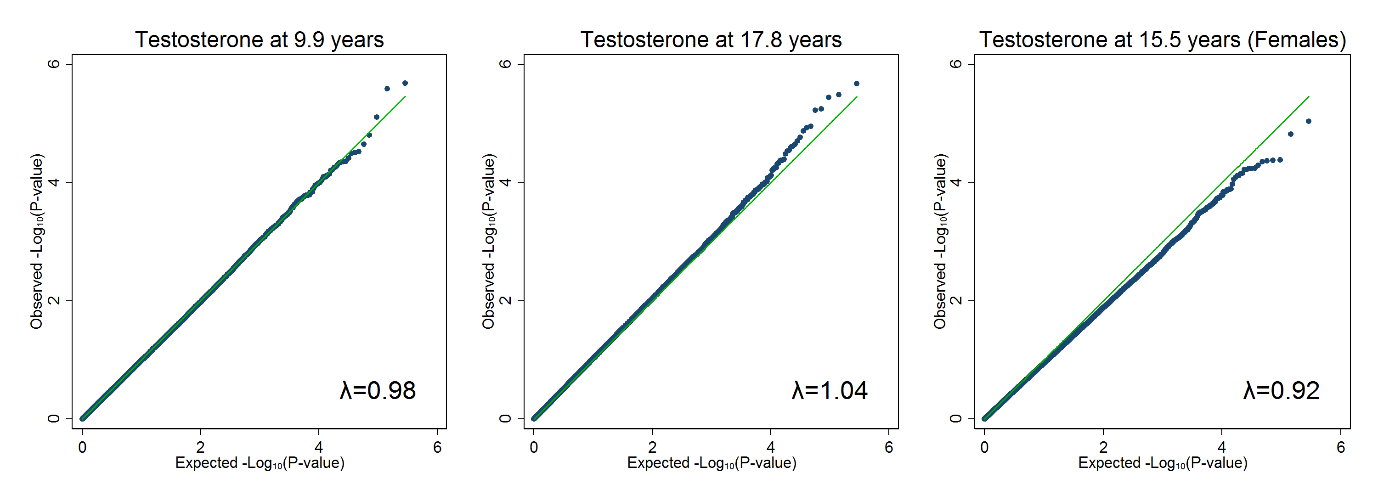
*

Figure S5 QQ plots of total testosterone EWAS at 9.9 and 17.8 years in males and 15.5 years in females.


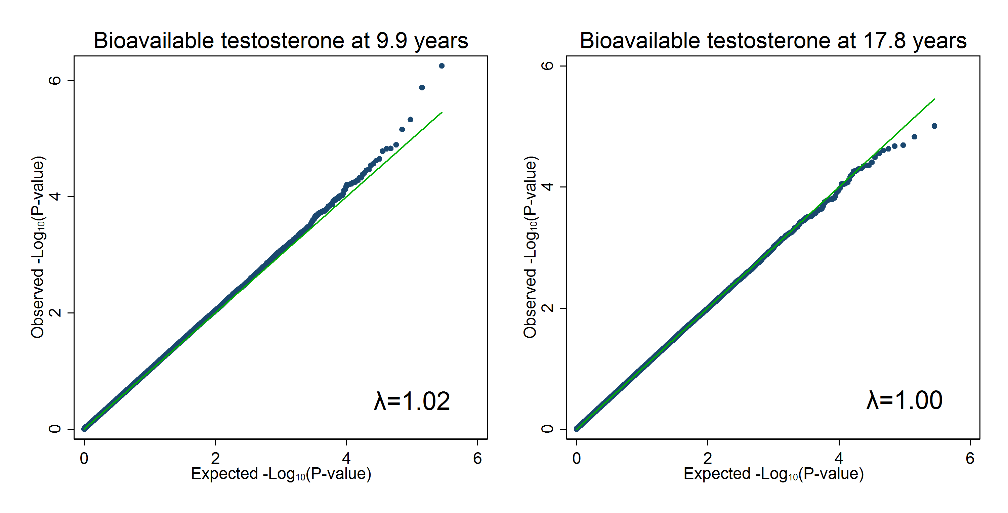


Figure S6 QQ plots of total testosterone EWAS at 9.9 and 17.8 years in males.


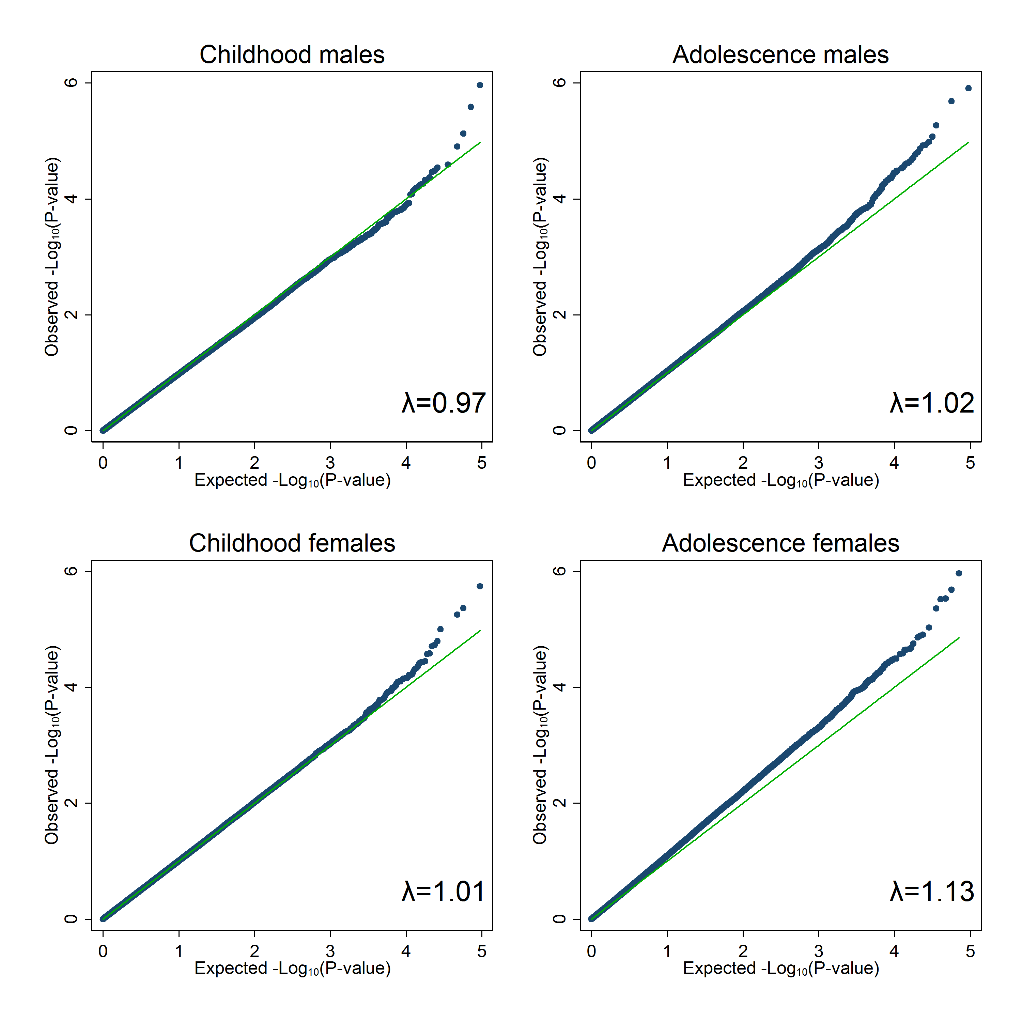


Figure S7 QQ-plots of EWAS models of rs12150660 in childhood and adolescence, stratified by sex.

## REFERENCES

1. Boyd A, Golding J, Macleod J, Lawlor DA, Fraser A, Henderson J, et al. Cohort Profile: The ‘Children of the 90s’—the index offspring of the Avon Longitudinal Study of Parents and Children. International Journal of Epidemiology. 2012;42(1):111-27.

2. Relton CL, Gaunt T, McArdle W, Ho K, Duggirala A, Shihab H. Data resource profile: Accessible Resource for Integrated Epigenomic Studies (ARIES). Int J Epidemiol. 2015;44.

3. Naeem H, Wong N, Chatterton Z, Hong MK, Pedersen J, Corcoran N, et al. Reducing the risk of false discovery enabling identification of biologically significant genome-wide methylation status using the HumanMethylation450 array. BMC Genomics. 2014;15(1):51.

4. Tukey J. Exploratory Data Analysis. Addison-Wesley. 1977:p43-4.

5. Leek JT, Johnson WE, Parker HS, Jaffe AE, Storey JD. The sva package for removing batch effects and other unwanted variation in high-throughput experiments. Bioinformatics. 2012;28(6):882-3.

6. Houseman EA, Accomando WP, Koestler DC, Christensen BC, Marsit CJ, Nelson HH. DNA methylation arrays as surrogate measures of cell mixture distribution. BMC Bioinformatics. 2012;13.

7. Aryee MJ, Jaffe AE, Corrada-Bravo H, Ladd-Acosta C, Feinberg AP, Hansen KD, et al. Minfi: a flexible and comprehensive Bioconductor package for the analysis of Infinium DNA methylation microarrays. Bioinformatics. 2014;30(10):1363-9.

8. Reinius LE, Acevedo N, Joerink M, Pershagen G, Dahlén S-E, Greco D. Differential DNA methylation in purified human blood cells: implications for cell lineage and studies on disease susceptibility. PLoS One. 2012;7.

9. Khairullah A, Cousino Klein L, Ingle SM, May MT, Whetzel CA, Susman EJ, et al. Testosterone Trajectories and Reference Ranges in a Large Longitudinal Sample of Male Adolescents. PLoS ONE. 2014;9(9):e108838.

10. Maisonet M, Calafat AM, Marcus M, Jaakkola JJ, Lashen H. Prenatal Exposure to Perfluoroalkyl Acids and Serum Testosterone Concentrations at 15 Years of Age in Female ALSPAC Study Participants. Environ Health Perspect. 2015;123(12):1325-30.
